# Kinematic evaluation of movement smoothness in golf: relationship between the normalized jerk cost of body joints and the clubhead

**DOI:** 10.1186/1475-925X-13-20

**Published:** 2014-02-26

**Authors:** Ahnryul Choi, Su-Bin Joo, Euichaul Oh, Joung Hwan Mun

**Affiliations:** 1 Department of Bio-Mechatronic Engineering, College of Biotechnology and Bioengineering, Sungkyunkwan University, 300 Chunchun, Jangan, Suwon, Gyeonggi 440-746, Republic of Korea; 2 College of Pharmacy, The Catholic University of Korea, Bucheon, Gyeonggi 420-743, Republic of Korea

## Abstract

**Background:**

When the human body is introduced to a new motion or movement, it learns the placement of different body parts, sequential muscle control, and coordination between muscles to achieve necessary positions, and it hones this new skill over time and repetition. Previous studies have demonstrated definite differences in the smoothness of body movements with different levels of training, i.e., amateurs compared with professionals. Therefore, we tested the hypothesis that skilled golfers swing a driver with a smoother motion than do unskilled golfers. In addition, the relationship between the smoothness of body joints and that of the clubhead was evaluated to provide further insight into the mechanism of smooth golf swing.

**Methods:**

Two subject groups (skilled and unskilled) participated in the experiment. The skilled group comprised 20 male professional golfers registered with the Korea Professional Golf Association, and the unskilled group comprised 19 amateur golfers who enjoy golf as a hobby. Six infrared cameras (VICON460 system) were used to record the 3D trajectories of markers attached to the clubhead and body segments, and the resulting data was evaluated with kinematic analysis. A physical quantity called jerk was calculated to investigate differences in smoothness during downswing between the two study groups.

**Results:**

The hypothesis that skilled golfers swing a driver with a smoother motion than do unskilled golfers was supported. The normalized jerk of the clubhead of skilled golfers was lower than that of unskilled golfers in the anterior/posterior, medial/lateral, and proximal/distal directions. Most human joints, especially in the lower body, had statistically significant lower normalized jerk values in the skilled group. In addition, the normalized jerk of the skilled group’s lower body joints had a distinct positive correlation with the normalized jerk of the clubhead with r = 0.657 (p < 0.01).

**Conclusions:**

The result of this study showed that skilled golfers have smoother swings than unskilled golfers during the downswing and revealed that the smoothness of a clubhead trajectory is related more to the smoothness of the lower body joints than that of the upper body joints. These findings can be used to understand the mechanisms behind smooth golf swings and, eventually, to improve golf performance.

## Background

Golf requires accuracy controlling the ball’s direction and flight distance [1]. The flight distance of the driver shot is one of the most important parts of the game because it sets the tone for the rest of the game and heavily influences the strategies for the following shots. In addition, the downswing phase takes place a considerable amount of energy consumption to generate high clubhead velocity [2]. Therefore, analyzing, understanding, and mastering the downswing can improve the overall game performance and management [3].

A golf swing involves complex and continuous rotational movements of each joint in the body, and the muscle contraction sequence and timing of the impact between the club and ball are important components of a successful swing [4]. Okuda et al. proposed that the sequential rotation of each joint involved in golf swing [5], called proximal-to-distal sequencing (PDS) [6], is the most important factor for successful golf shots. This series of movements builds momentum from the proximal to distal segments, and skilled golfers have been shown to be highly effective and efficient in these movements by a variety of studies [7-10]. Thus, a successful golf swing can be achieved by rotating the joints and harmoniously coordinating these movements.

A fast, accurate, consistent, and smooth movement has a high coupling in the body joints and segments and extends to successful performance [11]. According to Bril et al., skilled or dexterous action consists of smoothness, flexibility, precision, speed, adaptability, regularity, and optimization, and functionally coordinating these conditions is crucial [12]. Smoothness is achieved by purposefully repeating a movement and making necessary corrections to improve the motion [13]. The success of a human movement is judged by the smoothness of the motion, which can be quantified as jerk [14].

Jerk is defined as a change in acceleration rate over time and is the third derivative of displacement. The smoothest motion has the lowest jerk. There have been many attempts to describe the smoothness in a variety of movements. Hreljac compared the jerk in the heel of skilled middle- to long-distance runners to that of other athletes (from soccer or tennis) during running and fast walking [13]. The runners had significantly lower jerk than did other athletes, and Hreljac concluded that the runners tended to exhibit smoother movements than non-runners during both running and fast walking. In addition, by analyzing jerk, Yan et al. found that the arm movement involved in overarm throwing becomes smoother as one becomes an adult [14]. More recently, Sakata et al. studied the effect of age-related changes in the smoothness of lower body joints during lifting, and demonstrated high jerk values in the ankle and hip joints of older subjects, pointing to less smooth movements in this group [15].

An attempt to analyze the smoothness of golf putting was recently performed by Choi et al., who compared jerk among 3 groups: professional, recreational, and novice golfers [16]. They found a significant difference between the novice golfers and the other groups. Nevertheless, studies investigating the smoothness of golf swing movements are quite rare [16], and none have analyzed the smoothness of a driver swing.

In this study, using jerk, which quantitatively represents the smoothness of a motion, differences between skilled and unskilled golfers were analyzed during the downswing with a driver. We tested the hypothesis that the jerk of the clubhead during the driver downswing is lower in skilled golfers than in unskilled golfers. In addition, basic data to investigate the mechanism of a smooth clubhead movement was proposed by analyzing the correlations between the jerk of individual body joints and that of the clubhead.

## Methods

### Subjects & apparatus

Twenty skilled golfers and 19 unskilled golfers with no past history of musculoskeletal disease participated in this study. The skilled golfers were professional athletes (average career length 8.4 ± 5.0 years) who were all registered with the KPGA (Korea Professional Golf Association). The unskilled golfers were amateurs who do not play golf professionally. All subjects were right-handed and provided a written informed consent prior to experiments. This study was approved by the Institutional Review Board. Physical and swing characteristics of the skilled and unskilled golfers are presented on Table 1. There were no statistical differences between skilled and unskilled groups except for handicap scores (p < 0.01).

**Table 1:** Subject characteristics (Mean ± SD)

	**Skilled golfers**	**Unskilled golfers**	**Significance**
	**(Males, N = 20)**	**(Males, N = 19)**	
Age (years)	37.3 ± 9.1	40.3 ± 11.7	NS
Height (m)	173.8 ± 5.2	171.6 ± 5.7	NS
Weight (kg)	71.5 ± 12.0	71.8 ± 8.6	NS
Handicap (strokes)	< 0	16.7 ± 6.7	*p < 0.01*
Peak clubhead speed (m/s)	39.2 ± 4.6	36.3 ± 10.0	NS
Downswing duration (sec.)	0.307 ± 0.04	0.327 ± 0.04	NS

NS Non-significant.

**Figure 1:**
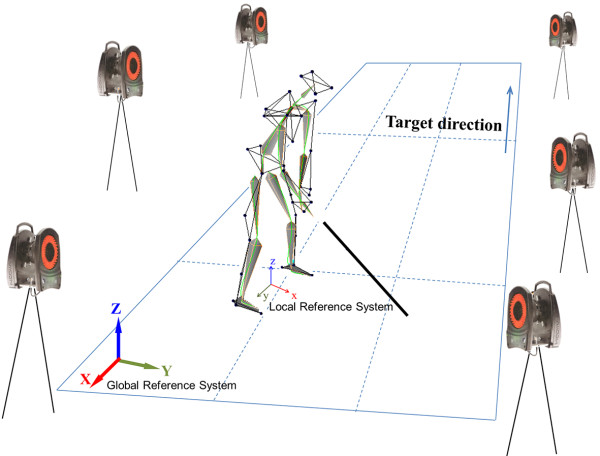
Golf swing analysis system.

Six infrared cameras (VICON460, Oxford Metrics, Oxford, UK) were used as measurement devices, and the sampling rate was set at 120Hz. The 3D coordinates were extracted from the 6 synchronized image coordinates. Figure 1 shows the overall experimental system.

### Experimental procedures

Thirty-five optical markers were attached to anatomical landmarks of the golfers. The placement of each marker was based on the modified Helen Hayes markerset protocol [17,18], and an additional marker was attached to the clubhead for the jerk analysis. Before the experiment, subjects completed a warm up with large dynamic movements and static stretches [19], and each subject was able to adapt to the laboratory environment with a practice swing. Each participant performed 3 swings, and the best shot, decided by how the participant felt about the shot and also by the quality of the reconstructed 3D data, was used in the analysis [20]. The analysis was limited to the downswing, which is the interval from the top of the backswing to the point of impact between the club and the ball. The top of the backswing was defined as the moment when the point of maximum clubhead rotation [20].

### Data & statistical analysis

**Figure 2:**
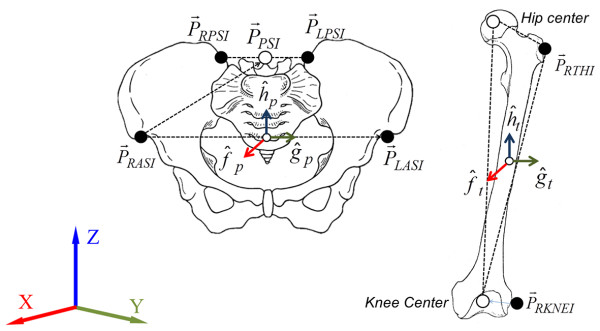
Anatomical reference system of the pelvis and femur.

To remove high-frequency noise, a zero leg, 4^th^-order, low-pass Butterworth filter was used, and the cut-off frequency was set between 10 and 20Hz through visual inspection of the frequency spectrum for each marker [21]. To calculate the joint rotation center of the body, the 3D marker trajectories were analyzed with SB-Clinic software (SWINGBANK Ltd, Korea), which is a golf swing analysis system. The kinematic model used in this system had 15 segments, 14 joints, and 36 degrees of freedom [22], and the 3D trajectory of the clubhead and rotation of body joints were calculated. Figure 2 and Equation 1–2 show the process of calculating the relative angular displacement of the hip joint. The anatomical reference system of each body segment was constructed from the coordinates of markers attached to the femur and pelvis (T_p_ and T_f_). The anatomical angular displacement of the each joint, which leads to the relative orientation of the proximal and distal segments, was calculated by using the Euler angle. The transformation matrix can be expressed as follows:

(1) $$T_{p}=\left[{\hat{f}}_{p}\;{\hat{g}}_{p}\;{\hat{h}}_{p}\right]=\left[\begin{array}{ccc} a_{11} & a_{12} & a_{13}\\ a_{21} & a_{22} & a_{23}\\ a_{31} & a_{32} & a_{33} \end{array}\right]\;,\;T_{t}=\left[{\hat{f}}_{t}\;{\hat{g}}_{t}\;{\hat{h}}_{t}\right]=\left[\begin{array}{ccc} b_{11} & b_{12} & b_{13}\\ b_{21} & b_{22} & b_{23}\\ b_{31} & b_{32} & b_{33} \end{array}\right]$$

(2) $${T_{t}}^{-1}T_{p}=\left[\begin{array}{ccc} \mathrm{c\varphi c\psi}-\mathrm{s\varphi c\theta s\psi} & -\mathrm{c\varphi c\psi}-\mathrm{s\varphi c\theta s\psi} & \mathrm{s\varphi s\theta}\\ \mathrm{s\varphi c\psi}-\mathrm{c\varphi c\theta s\psi} & -\mathrm{s\varphi s\psi}-\mathrm{c\varphi c\theta c\psi} & -\mathrm{c\varphi s\theta}\\ \mathrm{s\theta c\psi} & \mathrm{s\theta c\psi} & \mathrm{c\theta} \end{array}\right]$$

where c = cos and s = sin.

The trajectory of the clubhead was calculated from the local reference system based on the ball address posture. The origin of the local reference system was the midpoint between the center of the right and left ankle joints. The y-axis was the unit directional vector from center of the left to the right ankle joints, and the z-axis coincided with the global Z-axis. The x-axis was calculated as the cross-product of the y- and z-axes (Figure 1). The trajectory data of the marker attached to the clubhead was differentiated 3 times in the time domain and factored into the jerk endpoint, and the jerk in all 36 rotational degrees of freedom was obtained in the same way. To remove the influence of movement duration and distance, normalized jerk (NJ) was calculated with the following formula [14].

(3) $$\text{Normalized}\;\text{jerk}\left(\text{NJ}\right)=\sqrt{\frac{1}{2}\int \text{jerk}{\left(t\right)}^{2}\times \left(\frac{\text{duratio}n^{5}}{\mathrm{lengt}h^{2}}\right)\;\text{dt}}$$

A Mann-Whitney test was applied for comparative assessments of the skilled and unskilled groups, and correlation analysis was performed to define the relationship between the NJ of each human joint and that of the clubhead. All statistics were calculated using the SAS statistical analysis program (SAS version 9.1), and the significance level was set at p < 0.05.

## Results

### 3D trajectories of a clubhead between skilled and unskilled golfers

**Figure 3:**
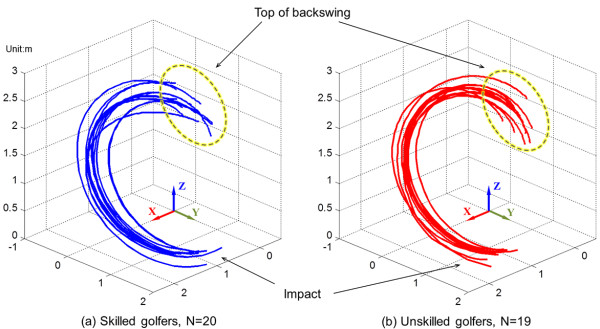
3D traces of a clubhead during the downswing in the skilled (a) and unskilled golfers (b).

Figure 3 shows 3D traces of the clubhead during the downswing of both skilled and unskilled golfers. The exact location of the top of the backswing differed for each participant; however, the 3D trajectory of the clubhead for all participants had a “C” shape as shown with no obvious differences between the groups.

### Clubhead jerk

**Figure 4:**
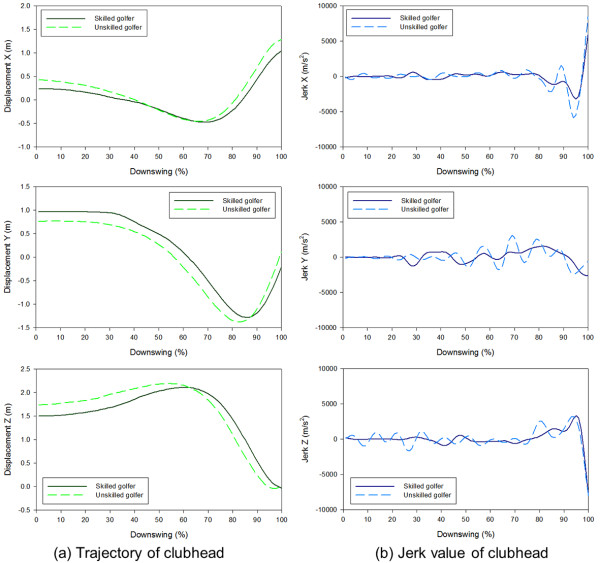
**Clubhead trajectory and jerk of representative skilled (a) and unskilled (b) golfers during the downswing.** The upper, middle and lower panels represent the values of x-, y- and z-axis of the local reference system respectively.

**Figure 5:**
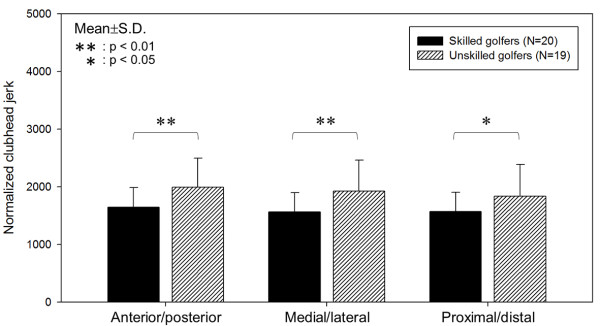
Comparisons of the NJ of the clubhead between skilled and unskilled golfers during the downswing.

Figure 4 shows the trajectory and jerk of the clubhead in each axis during the downswing in representative subjects from the skilled and unskilled groups. When jerk was taken into account, the unskilled golfers’ trajectories had more irregularities and bigger changes in jerk than did the skilled golfers’ trajectories. Neither the skilled nor unskilled group’s jerks had a distinct pattern. However, compared with skilled golfers, unskilled golfers had wider range of jerk values deviating from 0, as shown in the graph. Figure 5 demonstrates the NJ of the clubhead during the downswings of skilled and unskilled golfers. The NJ of the clubhead was significantly lower in each trajectory for skilled golfers.

### NJ of human joints

The NJ values for each joint of the skilled and unskilled golfers’ downswing are presented in Table 2. Generally, the skilled golfers had lower NJ values, and there were statistically significant differences between groups for most joints in the lower extremities. The number of joints with statistically significant differences between groups decreased moving from the ankles to the upper limbs. The right hip, right shoulder, right wrist, and left and right elbows had lower NJ values in unskilled golfers than in skilled golfers, but none of these differences were statistically significant (p > 0.05).

**Table 2:** Comparisons of the NJs of all joint angles between the skilled and unskilled golfers during the downswing

		**Skilled**	**Unskilled**	**p-value**
Left ankle	Flx/ext	215.0 ± 92	496.3 ± 242	<0.01**
	Abd/add	107.1 ± 57	240.3 ± 167	<0.01**
	Axial rot	105.9 ± 50	254.5 ± 124	<0.01**
Right ankle	Flx/ext	298.5 ± 109	459.7 ± 258	<0.01**
	Abd/add	194.0 ± 59	310.7 ± 185	<0.01**
	Axial rot	98.4 ± 40	177.5 ± 97	<0.01**
Left knee	Flx/ext	187.9 ± 84	324.9 ± 140	<0.01**
	Abd/add	162.7 ± 50	346.8 ± 108	<0.01**
	Axial rot	147.7 ± 49	288.5 ± 280	0.018*
Right knee	Flx/ext	297.1 ± 145	331.7 ± 129	NS
	Abd/add	237.7 ± 127	391.8 ± 219	<0.01**
	Axial rot	211.1 ± 124	241.6 ± 130	<0.01**
Left hip	Flx/ext	180.3 ± 60	216.7 ± 80	0.027*
	Abd/add	177.8 ± 47	222.1 ± 70	<0.01**
	Axial rot	280.4 ± 158	359.8 ± 204	NS
Right hip	Flx/ext	204.9 ± 88	169.3 ± 88	NS
	Abd/add	151.5 ± 70	237.8 ± 103	<0.01**
	Axial rot	343.6 ± 136	372.7 ± 183	NS
Spine	Flx/ext	115.4 ± 61	125.4 ± 43	NS
	Abd/add	282.4 ± 146	286.9 ± 125	NS
	Axial rot	197.4 ± 92	252.4 ± 177	NS
Neck	Flx/ext	275.7 ± 139	315.3 ± 184	NS
	Abd/add	300.4 ± 176	353.4 ± 143	NS
	Axial rot	105.9 ± 55	186.4 ± 222	NS
Left shoulder	Flx/ext	213.9 ± 151	227.1 ± 83	NS
	Abd/add	211.7 ± 134	247.4 ± 164	NS
	Axial rot	518.3 ± 142	728.9 ± 326	0.024*
Right shoulder	Flx/ext	215.8 ± 135	272.1 ± 166	NS
	Abd/add	312.0 ± 135	421.0 ± 48	0.029*
	Axial rot	320.5 ± 194	239.6 ± 100	NS
Left elbow	Flx/ext	455.3 ± 331	214.4 ± 85	NS
Right elbow	Flx/ext	329.5 ± 149	282.9 ± 100	NS
Left wrist	Flx/ext	480.4 ± 213	627.6 ± 466	NS
	Abd/add	635.5 ± 439	1120.4 ± 552	0.021*
Right wrist	Flx/ext	1951.9 ± 1442	1622.9 ± 1686	NS
	Abd/add	1211.3 ± 685	1241.7 ± 2197	NS

Flx/ext: Flextion/extention, Abd/add: Abduction/adduction, Rot: Rotation, *p < 0.05, **p < 0.01, NS: Non-significant.

### Correlation analysis between NJ of clubhead and human joints

**Figure 6:**
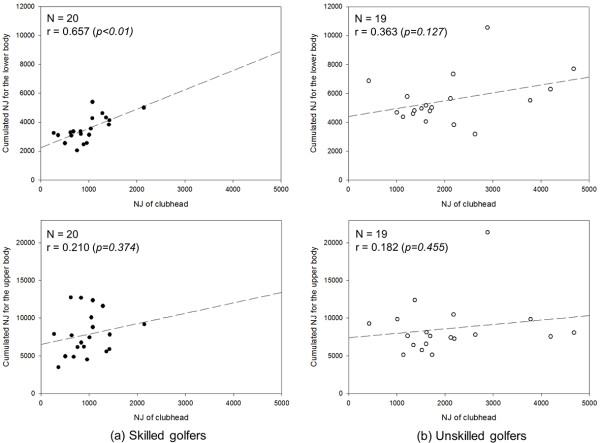
Correlation between the NJ of the lower (Top) and upper body (Bottom) and the clubhead in the downswing of the skilled (a) and unskilled golfers (b).

Figure 6 presents the correlation coefficients of the cumulated NJ for the different body joints and the clubhead during the downswing. A cumulative NJ of the lower body was calculated by adding the NJ values of the left and right ankles, knees and hips for all directions. A cumulative NJ of the upper body was calculated by adding the NJ values of the wrists, elbows, shoulder, neck, and spine for all directions. In the skilled group, the NJ of lower body was statistically significantly correlated with the NJ of the clubhead with a correlation coefficient of r = 0.657 (p < 0. 01). The correlation coefficient between the NJs of the lower body and the clubhead in the unskilled group was 0.363 (p = 0.127), which was not significant. In both the skilled and unskilled groups, the NJs of the upper body and the clubhead were not significantly correlated (r = 0.210, p = 0.374 for skilled golfers and r = 0.182, p = 0.455 for unskilled golfers).

Table 3 presents the results of the NJ correlation analysis for each joint and the clubhead during the downswing of skilled and unskilled golfers. The NJ in each joint was calculated by adding the jerks for each degree of freedom for a joint. Generally, the skilled golfers had more joints that were significantly correlated to the clubhead, and all lower body joints were positively correlated to the clubhead. Unskilled golfers also tended to have more significant correlations in the lower body than in the upper body, but the pattern was not as prevalent.

**Table 3:** **Correlation coefficient between the cumulative NJs of the joints and the NJ of clubhead during the downswing of skilled and unskilled golfers ( *p-value *)**

	**Skilled**	**Unskilled**
Left ankle	0.519 (0.019)*	0.195 (0.425)
Right ankle	0.558 (0.011)*	0.248 (0.307)
Left knee	0.528 (0.017)*	0.225 (0.353)
Right knee	0.549 (0.012)*	0.368 (0.121)
Left hip	0.474 (0.035)*	0.473 (0.041)*
Right hip	0.468 (0.038)*	0.509 (0.026)*
Spine	0.372 (0.106)	0.271 (0.261)
Neck	0.302 (0.196)	0.249 (0.305)
Left shoulder	0.367 (0.122)	0.426 (0.069)
Right shoulder	0.188 (0.426)	0.249 (0.303)
Left elbow	0.030 (0.898)	0.145 (0.552)
Right elbow	0.499 (0.025)*	0.212 (0.383)
Left wrist	0.195 (0.410)	0.040 (0.872)
Right wrist	0.075 (0.754)	0.095 (0.699)
Lower body	0.657 (0.002)**	0.363 (0.127)
Upper body	0.210 (0.374)	0.182 (0.455)

*p<0.05,**p<0.01.

## Discussion

On the basis of the general observation that experts or professionals seem to have smoother motions [13,14,16], this study was analyzed whether skilled golfers have a smoother golf driver swing than unskilled golfers. To quantify smoothness, jerk, the third derivative of displacement, and the NJ of the clubhead trajectory and of the rotational of all body joints during the downswing were calculated. Also, to better understand the mechanism of a smooth swing, the correlations between the NJs of each joint and the clubhead were calculated.

Since the jerk is the third differentiated trajectory value, random noise can accumulate. Jerk is reportedly sensitive to data smoothing methods [13]. Hreljac tried to minimize this potential error by using a double data smoothing method by filtering raw trajectory data and then filtering acceleration to calculate the jerk in heel movements during running and fast walking [23]. We tested a variety of cut-off frequencies and double data smoothing methods and selected a cut-off frequency for a low-pass filter by visually inspecting the frequency spectrum of each marker. Absolute differences jerk value depended on the data smoothing method, but relative differences between groups and the results of the statistical analysis were always consistent. Even though jerk is sensitive to data smoothing, the filter and cut-off frequency used in this study were appropriate for comparing the two groups.

High jerk values can be interpreted in 2 ways: strong muscles or decreased smoothness [15]. Puniello et al. proposed that the jerk for the vertical trajectory of box lifting is significantly and positively correlated with hip extensor strength [24]. Sakata et al. proposed that the jerk values of the ankle and hip joints increased because the smoothness of lower body joints was lower in the aged group [15]. In general, professional athletes are much stronger than amateurs, and according to previous studies, professional athletes have better muscle balance in the lower body, better weight shifts during movements, and more coordinated sequential muscle activation than amateurs [5,25]. In the current study, the skilled group had lower jerk in most joints and the clubhead, indicating smoother movements.

This study revealed that although 3D tracing of the downswing movement, when graphed, seemed similar between the skilled and unskilled groups (Figure 3), there was significant and noticeable differences between groups when jerk was taken into account (Figure 4). According to the Newton’s second law ‘F = ma’, force and acceleration have a proportional relationship when the mass is fixed. Therefore, jerk, the derivative of acceleration, can be defined as the variation of applied forces. The graph of jerk values (Figure 4) showed bigger fluctuation with wider distance between each peak and trough and steeper slopes, which implies an unbalanced force distribution to the club and unnecessary physical exertion during the downswing. The unskilled golfers exhibited higher NJ in most of joints and clubhead, which suggests less smoothness of movements due to inefficient motor control [26].

Another novel suggestion from this study is that the clubhead smoothness during the downswing is highly related to the smoothness of the lower body joints. Most previous literature dealing with golf swing mechanisms has analyzed the upper limbs [27,28]; only a few have studied the importance of the lower body. However, the fact that a robust lower body is required in upper body-oriented activities, such as pitching and hitting in baseball, is established and well accepted. Continuous movement of upper body joints, such as the PDS, is required in golf for a long drive, but the current study shows that smooth lower body movement is important for a smooth swing. Therefore, building a strong and durable lower body through adequate lower limb training is essential for controlling smoothness. Furthermore, future kinematic and kinetic studies are necessary to establish the precise mechanism that leads to a smooth clubhead trajectory.

The results of this study can be summarized as follows.

1. Skilled golfers had lower clubhead NJ in the golf driver downswing. This result implies that clubhead movements in skilled golfers were smoother than in unskilled golfers.

2. Skilled golfers had lower NJ in most joint than did unskilled golfers, although not all differences were statistically significant. The differences between groups for most lower body joints were statistically significant. The differences for most upper body joints were not statistically significant.

3. The NJs of the joints and the clubhead were positively correlated in the skilled group, and the cumulative NJ of the lower body had the highest correlation with the clubhead (r = 0.657, p < 0.01). The unskilled group did not have as strong of a correlation. This result can be used in future studies to investigate the mechanism behind smooth clubhead movement leading to smoother and more efficient golf swings.

This study confirmed that the jerk of the golf driver swing can be used as a quantitative measure to show differences in smoothness and that swing smoothness should be used in teaching golf. Nevertheless, this study has 2 limitations: only the downswing was analyzed and only the driver swing was analyzed. Future work should analyze the entire swing using various golf clubs. Fundamental and in-depth research on the mechanisms generating smoothness is also necessary.

## Competing interests

All authors declare that they have no competing interests.

## Authors’ contributions

Ahnryul Choi made substantial contributions to the data analysis and interpretation and was involved in drafting the manuscript. Su-Bin Joo conducted the experiments and data acquisition. Prof. Euichaul Oh contributed to the study concept. Prof. Joung Hwan Mun participated in the study design. In addition, Prof. Euichaul Oh and Joung Hwan Mun critically revised and gave final approval of the manuscript. All authors read and approved the final manuscript.
